# Plasmid-encoded NP73-102 modulates atrial natriuretic peptide receptor signaling and plays a critical role in inducing tolerogenic dendritic cells

**DOI:** 10.1186/1479-0556-9-3

**Published:** 2011-01-10

**Authors:** Weidong Zhang, Xueqin Cao, Dongqing Chen, Jia-wang Wang, Hong Yang, Wenshi Wang, Subhra Mohapatra, Gary Hellermann, Xiaoyuan Kong, Richard F Lockey, Shyam S Mohapatra

**Affiliations:** 1 Department of Internal Medicine, Division of Allergy and Immunology, University of South Florida College of Medicine, Tampa, FL 33612, USA; 2 Department of Molecular Medicine, University of South Florida College of Medicine, Tampa, FL 33612, USA; 3 James A. Haley Veterans Hospital Medical Center, Tampa, FL 33612, USA; 4 Department of Internal Medicine, Division of Endocrinology, University of South Florida College of Medicine, Tampa, FL 33612, USA; 5 Donald A. Adam Comprehensive Melanoma Research Center, Moffitt Cancer Center, 12902 Magnolia Drive, SRB-24324, Tampa, FL 33612, USA; 6 Department of Internal Medicine, Division of Translational Medicine, University of South Florida College of Medicine, Tampa, FL 33612, USA

## Abstract

**Background:**

Atrial natriuretic peptide (ANP) is an important endogenous hormone that controls inflammation and immunity by acting on dendritic cells (DCs); however, the mechanism remains unclear.

**Objective:**

We analyzed the downstream signaling events resulting from the binding of ANP to its receptor, NPRA, and sought to determine what aspects of this signaling modulate DC function.

**Methods:**

We utilized the inhibitory peptide, NP73-102, to block NPRA signaling in human monocyte-derived DCs (hmDCs) and examined the effect on DC maturation and induced immune responses. The potential downstream molecules and interactions among these molecules involved in NPRA signaling were identified by immunoprecipitation and immunoblotting. Changes in T cell phenotype and function were determined by flow cytometry and BrdU proliferation ELISA. To determine if adoptively transferred DCs could alter the *in vivo *immune response, bone marrow-derived DCs from wild-type C57BL/6 mice were incubated with ovalbumin (OVA) and injected i.v. into C57BL/6 NPRA-/- knockout mice sensitized and challenged with OVA. Lung sections were stained and examined for inflammation and cytokines were measured in bronchoalveolar lavage fluid collected from parallel groups of mice.

**Results:**

Inhibition of NPRA signaling in DCs primes them to induce regulatory T cells. Adoptive transfer of wild type DCs into NPRA-/- mice reverses the attenuation of lung inflammation seen in the NPRA-knockout model. NPRA is associated with TLR-2, SOCS3 and STAT3, and inhibiting NPRA alters expression of IL-6, IL-10 and TGF-β, but not IL-12.

**Conclusions:**

Modulation of NPRA signaling in DCs leads to immune tolerance and TLR2 and SOCS3 are involved in this induction.

## Introduction

Allergic asthma is a chronic inflammatory disease of the lung, involving an aberrant T helper-2 (Th2) immune response to allergens. The etiology of asthma is complex and involves a number of signaling molecules and pathways as well as environmental factors. Atrial natriuretic peptide (ANP) is a cardiac hormone that regulates blood pressure and volume and the sodium/potassium balance. Atrial natriuretic factor is synthesized as a prohormone that is cleaved into a C-terminal peptide, ANP, and a group of three N-terminal peptides which are released into the circulation and may negatively inhibit ANP activity. ANP binds to a cell surface receptor, natriuretic peptide receptor A (NPRA), which is found on cells in the lung and airways as well as kidney and other tissues. Hormone binding to NPRA is the predominant trigger in the natriuretic system and generates the intracellular signaling molecule cyclic guanosine monophosphate (cGMP) which can activate cGMP-dependent protein kinase and initiate a cascade of events. Patients with asthma or inflammatory lung disease have elevated levels of circulating ANP [1], which suggests that manipulation of NPRA signaling might provide a therapeutic benefit for asthmatics [2].

The natriuretic peptide family comprises atrial natriuretic peptide, ANP, brain natriuretic peptide, BNP, C-type natriuretic peptide, CNP, *Dendroaspis *natriuretic peptide, DNP, and urodilatin [3]. The activities of ANP and BNP are similar, and their biological actions, such as vasodilation and natriuresis, are mediated through binding to their receptor, NPRA, which leads to production of the intracellular second messenger cGMP [4]. Besides expression in heart atria, ANP is also produced in various lymphoid organs [5], and the NPRA receptor is found on immune cells of numerous species highlighting the importance of NPRA signaling in the immune response [6].

Over-production of ANP can affect the adaptive immune system by altering dendritic cell (DC) differentiation and promoting a Th2 response characteristic of allergic disease [7]. However, the mechanism by which NPRA signaling in DCs alters the innate and adaptive immune responses is unclear. In animal models of allergic lung inflammation, we showed that ANP signaling through NPRA promotes lung histopathology [8]. In studying the regulation of NPRA, we discovered a peptide, NP73-102, from the N-terminus of the ANP prohormone that acts as a brake on ANP signaling by reducing expression of NPRA. NP73-102 consists of amino acids 73 to 102 of the ANP prohormone and has bronchoprotective effects in a mouse model of asthma and anti-inflammatory activity in human epithelial cells [9]. The amino acid sequence of this peptide is different from ANP and NP73-102 does not bind to NPRA and prevent ANP from attaching. NP73-102 reduces ANP-induced signaling by downregulating its receptor and by feedback inhibition of ANP production [10].

DCs express abundant NPRA while macrophages do not. It was therefore hypothesized that the effects of NPRA signaling on innate and adaptive immunity occur through NPRA-mediated alterations in gene expression in DCs. Little is known about the role of NPRA signaling in innate immunity and about the downstream effects of NPRA-mediated immunoregulation in DCs. Tolerogenic DCs present antigen to T cells, but do not deliver the signals for effector T-cell activation and proliferation. This lack of costimulation can result in T-cell apoptosis [11], T-cell anergy [12] or differentiation into regulatory T-cells (Tregs) [13]. The identification of ANP's effects on DCs as key regulators of peripheral tolerance to allergens may be important in the prevention and treatment of allergic diseases [14,15].

In this study, the NPRA inhibitory peptide NP73-102 was utilized to block NPRA signaling in human DCs and to analyze the downstream cascade events. The results demonstrate that Toll-like receptor-2 (TLR2) and suppressor of cytokine synthesis-3 (SOCS3) are key players in integrating NPRA signaling with innate immunity and in the induction of tolerogenic DCs.

## Materials and methods

### Isolation, transfection and viability assay of human dendritic cells

Human monocytes were isolated from peripheral blood mononuclear cells using the Monocyte Isolation Kit II (Miltenyi Biotec, Auburn, CA). Human monocyte-derived DCs (HmDCs) were generated from these cells as previously described [16]. Briefly, monocytes isolated from peripheral blood mononuclear cells were induced to differentiate into DCs by incubation with 200 ng/ml IL-4 and 50 ng/ml GM-CSF. Five day-old DCs were transfected with the indicated plasmid (3 μg/10^6 ^cells) using Lipofectamine 2000 (Invitrogen, Carlsbad, CA). After 24 hr, cell viability was measured by MTT assay (Sigma/Aldrich, St Louis, MO). Additional information on the viability assay is provided in Additional File 1.

### Cytokine measurement

Transfected hmDCs (1 × 10^6 ^cells/well) were cultured in 24-well plates for 24 h. Cytokine levels in the cell-culture supernatants were measured using a cytokine bead array kit (BioSystem, Bio-Rad) following the manufacturer's directions. All samples were assayed in duplicate.

### Isolation of naïve T cells, generation of Tregs and T-cell suppression assay

Human allogeneic naïve CD4+CD25-T cells were purified from umbilical cord blood using the naïve T cell isolation kit with biotinylated CD25 antibody (Miltenyi Biotec, Auburn, CA) and co-cultured with irradiated transfected hmDCs (10:1 ratio) in 24-well plates for 6 days. Expression of the Treg protein FoxP3 by co-cultured T cells was quantitated by flow cytometry (BD-FACScan, BD Biosciences, San Jose, CA). Also, total RNA was extracted and analyzed by reverse transcriptase-PCR for FoxP3. Tregs were purified with the Treg cell isolation kit (Miltenyi Biotec.) and a co-culture suppression assay was performed using a BrdU proliferation ELISA kit (Roche, IN, USA) as previously described [17]. Additional information is provided in the Additional File.

### Measurement of intracellular cGMP

Five day-old hmDCs (10^6 ^cells/sample) were transfected with the indicated plasmid and incubated in medium for 18 h. After incubation, the cells were removed from the plate, pelleted by centrifugation (750× g, 5 min) and intracellular cGMP in the cell pellets was measured with a cGMP ELISA kit (R & D Systems, Minneapolis, MN).

### Phagocytosis assay

Five day-old hmDCs (10^6^) were harvested 24 hr post-transfection and resuspended in RPMI 1640 medium supplemented with 2% FBS. The FITC-dextran phagocytosis assay was performed as described [18].

### Immunoprecipitation and immunoblotting

Five day-old hmDCs were transfected with 3 μg each of expression plasmid encoding NPRA, TLR2, STAT3 and SOCS3 and then harvested 24 hr post-transfection. Lysates (400 μg of protein/sample) were immunoprecipitated with antibody against TLR2, STAT3, SOCS3 or NPRA overnight at 4°C. The antibody complexes were precipitated by the addition of recombinant protein G agarose (Invitrogen, Carlsbad, CA). Eluted proteins were resolved on 12% SDS-PAGE gels, transferred to PVDF membranes (Bio-Rad, Hercules, CA) and immunoblotted with the indicated antibodies.

### Luciferase assay

HmDCs were transfected with the indicated plasmid and 48 h later, the cells were analyzed for luciferase activity. Additional detail is provided in the Additional File.

### Studies in mice: OVA sensitization, DC isolation and adoptive transfer

Bone marrow cells were removed from C57BL/6 NPRA-/- or wild type (WT) mice and cultured for 8 days as described previously [19]. Bone marrow-derived DCs (bmDCs) were purified using CD11c microbeads (Miltenyi Biotec, Auburn, CA), incubated with ovalbumin (OVA; 0.5 mg/ml) for 24 hr and injected i.v. into NPRA-/- mice (5 × 10^6 ^bmDCs/mouse), which had been sensitized (i.p) and challenged (i.n) with OVA (25 μg). Mice were euthanatized, lungs were lavaged with 1 ml of PBS, and BAL cytokines were quantitated by cytokine bead array (BioSystem, Bio-Rad, Hercules, CA). Lung histopathology was assessed using a previously described scoring system [20]. Additional details are provided in the Additional File.

### Statistical analysis

The results are expressed as means ± SEM. Data were analyzed using an unpaired two-tailed Student's *t *test.

## Results

### NP73-102 inhibits NPRA signaling in human DCs

**Figure 1:**
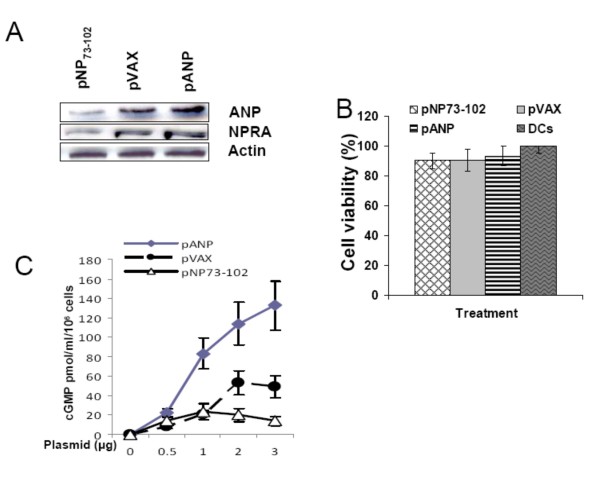
**NP73-102 is an inhibitor of NPRA signaling in hmDCs**. **(A) **HmDCs were transfected with the indicated plasmids. The cells were collected 24 hr after transfection, lysed and immunoblotted with the indicated antibodies. **(B) **Cell viability of hmDCs by MTT assay 24 hr after plasmid transfection. **(C) **HmDCs were transfected with different doses of the indicated plasmids. The cells were collected 18 hr after transfection and intracellular cGMP was measured by ELISA. Analyses shown are representative of two or three independent experiments.

Both endogenous ANP and NPRA are expressed by cultured hmDCs (Figure 1A). Transfection of hmDCs with pNP73-102, however, decreases the expression of endogenous ANP and NPRA compared to controls (Figure 1A). To determine if NP73-102 overexpression caused cytotoxicity in DCs, cell viability was measured 24 hr post-transfection. NP73-102 did not significantly affect cell viability at the dose used (Figure 1B). Intracellular cGMP levels in transfected hmDCs were also measured at 18 hr post-transfection. NP73-102 dose-dependently decreased cGMP production by blocking the activity of endogenous ANP compared to controls (Figure 1C), indicating that NP73-102 inhibits NPRA signaling in human DCs. The transfection efficiency of hmDCs transfected with pEGFP, as assessed by fluorescence microscopy of green fluorescent protein, was 41.3%.

### Inhibiting NPRA signaling alters cytokine production in human DCs

**Figure 2:**
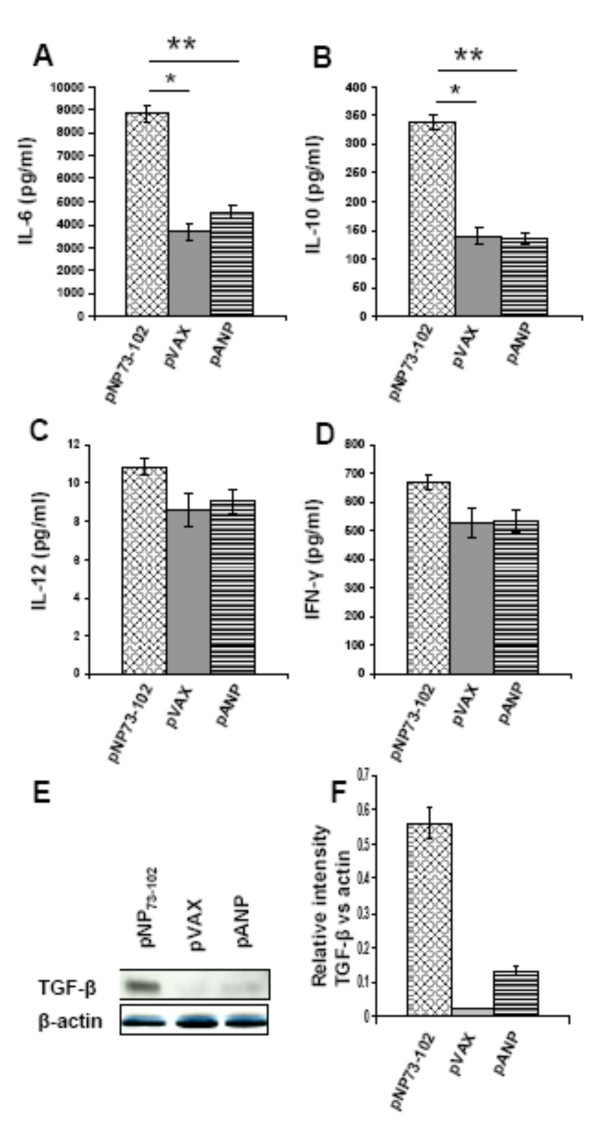
**NP73-102 inhibition of NPRA signaling alters hmDC cytokine profile**. **(A-D) **Transfected hmDCs were cultured in complete medium in 24-well plates for 24 hr. Cytokine levels in supernatants were measured in duplicate by cytokine bead array assay. The values are means ± SEM. *p < 0.05 and **p < 0.05 for NP73-102 vs pVAX and pANP respectively. **(E) **Immunoblotting for TGF-β expression in transfected DCs. **(F) **Protein bands were scanned and band density was quantitated using the Scion Image program. These results are from three separate experiments.

As demonstrated in Figure 1A, hmDCs produce endogenous ANP that can be down-regulated by NP73-102. Activation of the ANP-NPRA signaling pathway can alter cytokine profiles in hmDCs and inhibiting this pathway with pNP73-102 allowed greater production of IL-6, IL-10 and TGF-β (Figure 2A &2B, E &2F). IL-12 and IFN-γ levels did not change significantly among the groups (Figure 2C &2D).

### Inhibiting NPRA signaling in human DCs induces Tregs

**Figure 3:**
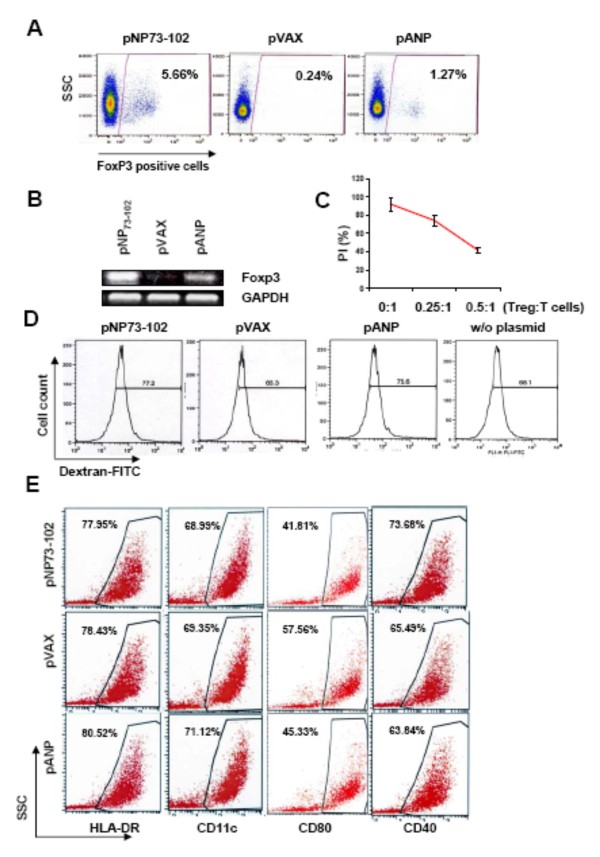
**Modulation of NPRA signaling in human monocyte-derived dendritic cells (hmDCs) alters Treg generation *in vitro***. **(A) **Flow cytometry assay for Foxp3-positive T cells in naïve CD4+ CD25-T cells co-cultured with plasmid-transfected hmDCs. Results shown are from one representative experiment of three repeats. **(B) **RT-PCR analysis of Foxp3 expression in naïve CD4+ T cells after co-culture with plasmid-transfected hmDCs. **(C) **Autologous CD4+CD25-T cells were co-cultured with NP73-102-induced CD4+CD25+ T cells at different ratios and the proliferation index (PI) was calculated. **(D) **Phagocytosis by hmDCs transfected with the various plasmids. DCs were harvested one day after transfection, incubated with FITC-dextran for 1 h at 37°C and counted by flow cytometry. For each sample, the background (mean value of fluorescence of cells exposed to FITC-dextran at 4°C) was subtracted from the mean value of fluorescence of hmDCs incubated at 37°C. **(E) **Flow cytometric analysis of phenotypic markers of transfected hmDCs. Results shown are from one representative experiment of three repeats.

Since blocking NPRA signaling by transfection of hmDCs with pNP73-102 up-regulates IL-10 and TGF-β expression, the role of NPRA in Treg induction was investigated. HmDCs were transfected with pNP73-102, pANP or pVAX and cocultured with allogeneic naïve CD4+ CD25-T cells (FoxP3-negative by RT-PCR). NP73-102 induced more FoxP3 expression than did ANP or vector alone (Figure 3A &3B). NP73-102-induced CD25+ T cells were able to suppress proliferation of CD4+CD25-T-cells in a dose-dependent manner (Figure 3C), suggesting that CD4+CD25+ T cells with suppressive ability can arise from CD4+CD25-naïve T cells co-cultured with NP73-102-treated hmDCs, correlating with the expression of FoxP3.

### Inhibiting NPRA signaling does not affect maturation of human DCs

The degree of hmDC maturation affects their capacity for Treg generation, and therefore the NPRA signal blockade was investigated to see if it alters hmDC maturation. Using the ability to phagocytose as a measure of maturation, pVAX or mock transfection slightly lowered hmDC phagocytosis compared to pNP73-102 or pANP (Figure 3D). Further, the hmDC phenotype analysis showed that HLA-DR, CD11c, CD40 and CD80 did not significantly change in expression among the groups (Figure 3E), suggesting that NPRA signaling does not affect hmDC maturation.

### NPRA inhibition alters expression of TLR-2, STAT3 and SOCS3 in human DCs

**Figure 4:**
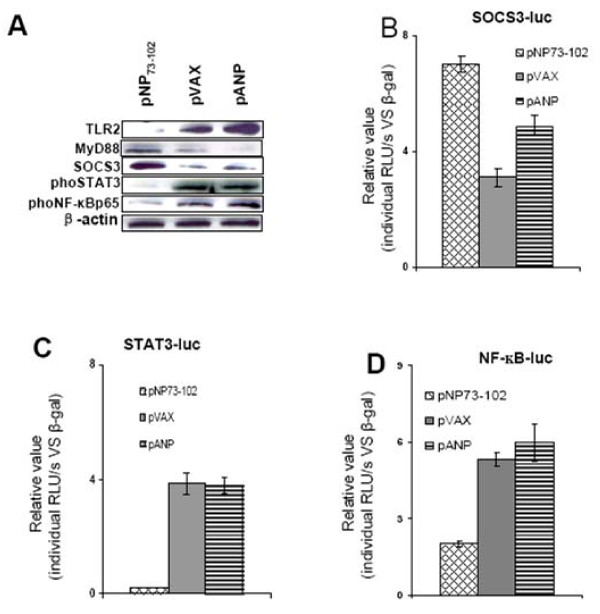
**Natriuretic peptides modulate the expression **of TLR2, STAT3 and SOCS3 in hmDCs**. (A) **HmDCs were transfected with the indicated plasmids, and cells were collected 24 hr after transfection for protein immunoblotting. **(B-D) **SOCS3, STAT3 and NF-κB reporter activities in DCs were measured after natriuretic peptide stimulation. The results shown are representative experiments from three independent assays.

IL-6 operates through the JAK1-STAT3 pathway, and triggering TLRs on mouse DCs can induce SOCS1 and SOCS3 in a STAT-dependent manner [21]. Results show that transfection with pNP73-102 down-regulated the level of activated phospho-STAT3 protein and enhanced expression of SOCS3 (Figure 4A), but not SOCS1 (*data not shown*). pNP73-102 selectively decreased TLR2 expression on DCs while elevating MyD88 compared to pANP or pVAX. We also found that pNP73-102 reduced the NF-κB transactivation from a promoter construct in hmDCs (Figure 4A). To confirm this effect, a luciferase reporter system was utilized to test the effect of NPRA inhibition on promoter activity of some target genes. As shown in Figure 4B-D, NP73-102 attenuated the activity of STAT3/NF-κB and increased the activity of SOCS3 in hmDCs.

### Protein interactions in the NPRA signal pathway

**Figure 5:**
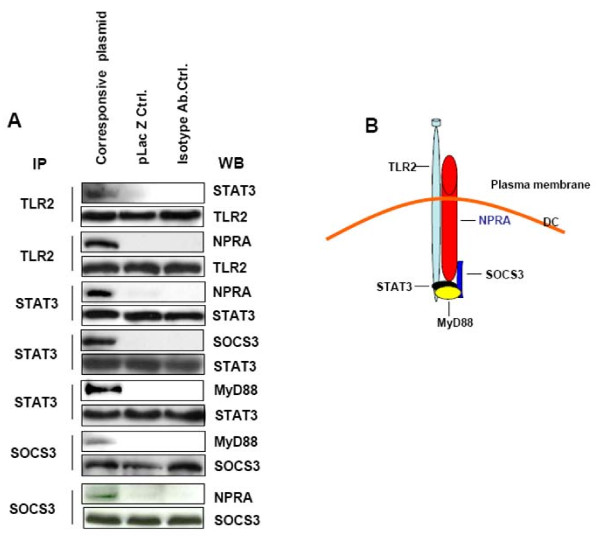
**Protein interaction analysis**. **(A) **HmDCs were harvested one day after transfection with the indicated plasmid. Cell lysates were immunoprecipitated (IP) with antibody against TLR2, STAT3, SOCS3 or NPRA and immunoblotted (IB) with STAT3, SOCS, MyD88, or NPRA antibodies, respectively. A representative experiment from three independent assays is shown. **(B) **Diagram of hypothesized protein interactions in the immune response associated with NPRA signaling in hmDCs.

This set of experiments was designed to determine which of the proteins that are known to be involved in DC function might be associated with NPRA. The hmDCs were transfected with NPRA, TLR2, STAT3 and SOCS3 expression plasmids, allowed to express for 24 hr, then whole-cell lysates were immunoprecipitated with the indicated antibody. The levels of expression of each of the proteins in the transfected cells were approximately the same as shown by the second set of bands in each IP. Precipitates were recovered and eluted proteins were separated by SDS-PAGE and immunoblotted for the indicated protein (first set of bands). The blots showed that NPRA was strongly pulled down with TLR2 and STAT3 and weakly with SOCS3 (Figure 5A). The corresponding bidirectional pull-down assays were done and showed similar results (*data not shown*). TLR2 was bound to STAT3, and the adaptor protein MyD88 bound both STAT3 and SOCS3 (Figure 5A). The IP data suggest that the immunoregulation of NPRA signaling in hmDCs may involve a specific interaction among these four proteins (Figure 5B). The tentative model shows one possible configuration that fits the observed association data, but further work is needed to clarify whether ANP binding to NPRA influences TLR2 activation or vice versa and how NP73-102 might alter the protein interactions.

### Adoptively transferred wild type mouse bone marrow-derived DCs (bmDCs) restore lung inflammation in NPRA-/- mice

**Figure 6:**
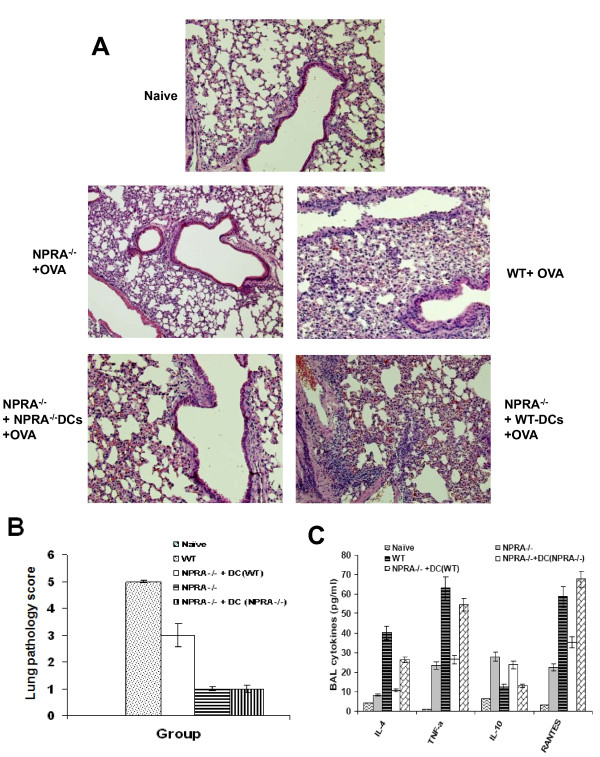
**Adoptive transfer of **bone marrow-derived DCs (bmDCs) **from WT mice increases inflammation in NPRA-/- mice**. Eight day-cultured bmDCs generated from WT or NPRA-/- C57BL/6 mice were incubated with OVA for 24 h and injected i.v. into C57BL/6 NPRA-/- mice (5 × 10^6 ^DCs/mouse) that had been sensitized and challenged with OVA. On day 8 after adoptive transfer, mice were sacrificed and lung sections were stained with H & E and examined under the microscope for histopathology (**A**). Lung pathology was also scored according to a 1-5 severity scale (**B**). Cytokines were measured by cytokine bead array assays in BAL fluid collected from parallel groups of mice (**C**). Data shown are representative of two experiments with similar results.

Since hmDCs express NPRA [7] and NP73-102 prevents NF-κB activation in hmDCs *in vitro *(Figure 4A, D), we reasoned that alterations in NPRA signaling in DCs should affect inflammation *in vivo*. To test this hypothesis, OVA-treated bmDCs from NPRA-/- or WT C57BL/6 were adoptively transferred into NPRA-/- mice that had been sensitized and challenged with OVA. As shown in Figure 6A, the lungs from NPRA-/- mice given bmDCs from wild-type mice exhibited inflammation similar to that of WT; however, OVA-treated NPRA-/- mice given no bmDCs or given bmDCs from NPRA-/- mice had little lung inflammation, suggesting that NPRA signaling in DCs plays a critical role in allergic lung inflammation. Inflammatory scoring by blinded observers of lung sections under the microscope (Figure 4B) confirmed the visualized results of Figure 4A. Cytokines were measured in BAL fluid collected from parallel groups of mice, and the results show that NPRA-/- mice given no bmDCs or given bmDCs from NPRA-/- mice have decreased expression of IL-4, TNF-α and RANTES, and increased IL-10 expression compared to other groups (Figure 6C).

## Discussion

A novel mechanism for inducing tolerogenic DCs by inhibition of NPRA signaling in DCs is described. Our demonstration that down-regulation of NPRA levels and reduction in NPRA signaling in DCs increases the population of Tregs may have important applications in treating respiratory disease and inflammatory conditions. Effective allergen immunotherapy involves generation of Treg cells, and targeted NPRA down-regulation may be used as a means to develop tolerogenic DCs that induce Tregs to ameliorate or prevent inflammation.

The discovery of the NPRA inhibitory peptide NP73-102, which reduces expression of NPRA and inhibits NPRA signaling and the activation of several pro-inflammatory transcription factors in epithelial cells [8,9], has provided the impetus to study the mechanism of how NPRA signaling affects inflammation and immunity. Increased NPRA signaling in DCs leads to a Th2 response, which restricts Treg production, while inhibiting signaling induces more IL-10 and TGF-β production and stimulates Treg formation.

Using an *in vitro *T cell-DC coculture system, down-regulation of NPRA by NP73-102 resulted in greater DC-mediated generation of Tregs. Analysis of DC surface marker expression and *in vitro *phagocytosis demonstrated that DC maturation was not significantly affected by NPRA signaling blockade. The mechanism underlying the NP73-102-induced Treg response is not fully understood. It may be due to inactivation of NF-κB activity by NP73-102 inhibition of NPRA signaling, since inhibition of NF-κB in DCs enhances their tolerogenic activity and prevents detrimental autoimmune diseases [22].

These data demonstrate that pNP73-102 increases the level of IL-10 and TGF-β, but not of IL-12 and IFN-γ, compared to cells given pANP or vector alone. TGF-β, which inhibits Th1 and Th2 development, is critical in Th17 development, in combination with IL-6 [23] and leads to the generation of Foxp3-positive regulatory T cells [24]. However, there was no increase in the number of Th17 lymphocytes, even in the presence of IL-6, in the DC-naïve T cell co-culture system, suggesting that Tregs may inhibit Th17 generation or that different cytokine profiles may produce diverse outcomes [25]. As shown in Figure 3A, the pANP-treated group also induced few Tregs versus the empty vector control, suggesting that TGF-β may be involved in the process (Figure 2E &2F). However, attenuation of NPRA signaling by pNP73-102 induced a greater amount of SOCS3 and generated more Tregs which supports the hypothesis that ANP signaling effects are mediated through production of tolerogenic DCs. Data show that ANP inhibits TGF-β-induced Smad2 and Smad3 nuclear translocation in rat pulmonary arterial smooth muscle cells [26], which is consistent with our finding that ANP induced less TGF-β production in human DCs than NP73-102.

The SOCS3 protein was strongly induced by both IL-6 and IL-10. SOCS3 selectively inhibits IL-6 signaling via its binding to the IL-6 receptor, but does not inhibit the IL-10 receptor [27]. The suppressive effect of SOCS3 is primarily restricted to STAT3 [28], and these results show that pNP73-102 inhibits STAT3 activity and enhances SOCS3 expression. This is in marked contrast to pANP and the vector control, which induce STAT3 phosphorylation and decrease SOCS3 expression in hmDCs. These data further support the idea that in this model, NPRA signaling in tolerogenic DCs involves the regulation of SOCS3 expression and STAT3 activity.

Cells exposed to pNP73-102 selectively diminished TLR2 expression compared to cells given pANP or vector. This could be explained by decreased NF-κB-mediated down-regulation of TLR through a binding site for NF-κB on the TLR2 promoter [29]. Reports have indicated that down-regulation of both TLR4 and TLR2 expression in mice decreases the expression of inflammatory cytokines and enhances production of anti-inflammatory cytokines, which induce immune tolerance [30]. Significantly, enhanced MyD88 expression was found in DCs treated with pNP73-102 compared to pANP and vector control. An LPS-inducible MyD88 is defective in its ability to induce IRAK phosphorylation and behaves as a dominant-negative inhibitor of LPS-induced NF-κB activation [31]. Also, *MyD88*-knockout mice show significantly reduced expression of SOCS3 [32], which is in part consistent with our data, although it is unclear why pANP induced higher SOCS3 expression in the absence of MyD88 than vector control. Thus, the enhanced expression of both SOCS3 and MyD88 in DCs may be associated with a reduced response to ANP, whereas the specific enhancement of SOCS3 and/or MyD88 expression may explain the generation of tolerogenic DCs.

Indirect inhibition of JAKs due to the binding of SOCS to membrane proximal regions of receptor chains results in steric hindrance of constitutive JAK binding to the receptor [33]. Inhibition of NPRA signaling by pNP73-102 through SOCS3 might occur by this mechanism. The IP data support this hypothesis. NPRA binds to SOCS3, and this interaction might contribute to the effects of NPRA signaling on immunoregulation. NPRA also binds to TLR2 and STAT3. However, TLR2 and SOCS3 involvement in regulation of NPRA expression (*unpublished data*) might result from these protein interactions rather than STAT3 involvement. Further work is needed to clarify whether the interactions among these proteins are direct or indirect.

In our animal model, we found that NPRA-/- mice had decreased expression of Th2-like cytokines, and that adoptive transfer of DCs from WT to NPRA-/- mice restored levels of these cytokines to those seen in WT. This is an important finding since it complements the *in *vitro results in an animal model. The lungs from NPRA-/- mice given DCs from WT mice exhibited inflammation similar to that of the WT. OVA-treated NPRA-/- mice given no DCs or given DCs from NPRA-/- mice did not have significant lung inflammation (Figure 6A, B), suggesting that DCs are the key mediators in modulating lung inflammation by NPRA signaling.

Taken together, our results demonstrate a novel mechanism for integration of TLR2, STAT3 and SOCS3 with NPRA signaling to regulate the immunomodulatory activity of DCs. They support the hypothesis that inhibition of NPRA signaling and TLR2 expression in DCs induces more IL-10 and TGF-β secretion and increases SOCS3 expression, thereby promoting the generation of Treg cells.

## Competing interests

The authors declare that they have no competing interests.

## Authors' contributions

WZ: contributed equally to the acquisition and analysis of data. XC contributed equally to the acquisition of data. DC, WW, HY and XK provided technical support. JWW contributed to the discussion. SM reviewed the manuscript and data presentation. GH prepared and edited the manuscript. RFL reviewed the manuscript. SSM directed research, reviewed the manuscript. All authors have read and approved the final manuscript.

## Supplementary Material

Additional file 1 **Additional file 1**. This supplementary material contains additional information about the viability assay, the T cell suppression assay, the luciferase assay, and the experiments in mice. Click here for file
