# Supplementary material for: Plasmid-encoded NP73-102 modulates atrial natriuretic peptide receptor signaling and plays a critical role in inducing tolerogenic dendritic cells
Source: Genet Vaccines Ther. 2011 Jan 10;9:3. doi: 10.1186/1479-0556-9-3 (PMC3025824; doi:10.1186/1479-0556-9-3)
Supplement: Additional file 1 — This supplementary material contains additional information about the viability assay, the T cell suppression assay, the luciferase assay, and the experiments in mice. [file 1479-0556-9-3-S1.DOC]

**Additional File**

**Determination of cell viability**

Five day-old hmDCs were transfected with the indicated plasmid (3 μg/10^6^ cells) in a volume of 200 μL and 24 hr later, 20 μL of MTT 3-[(4,5-dimethyl-thiazol-2yl)]-2,5-diphenyl tetrazolium bromide) stock solution (5 mg/ml, Sigma) was added to each tube and cells were incubated at 37°C for 3 hr. The MTT solution was removed by centrifugation. The cell pellets were resuspended in 200 μL of dimethyl sulfoxide (DMSO) and shaken vigorously for 20 min. The absorbance at 570 nm was measured and background absorbance at 690 was subtracted. The mean A_570_ value for cells not transfected with plasmid (negative control) was used to represent 100% cell viability. The individual A_570_ values of transfected cells were divided by the negative control value and expressed as a percent to determine the change in cell viability caused by each treatment.

***In vitro* T-cell suppression assay**

NP73-102-induced CD4+CD25+ Tregs were purified using a CD4+CD25+ regulatory T cell isolation kit (Miltenyi Biotec.) and co-cultured at the indicated ratios with autologous CD4+CD25- T cells for 72 hr in 96-well flat-bottom plates at 10^5^ cells/100 µl. Induced CD4+CD25+ T cells alone were cultured in parallel. Cells were activated with 2 µg/ml anti-CD3 and anti-CD28 antibodies in the presence of 10 ng/ml human recombinant IL-2. During the final 20 hr, 10 µl/100 µl BrdU labeling solution was added to the culture. The absorbance at 450 nm of each well was measured with an ELISA plate reader and background absorbance at 630 nm was subtracted. The proliferation index (PI) was calculated using the following equation:

A_450_ (T cells + Treg) – A_450_ Treg

PI = ----------------------------------------------

A_450_ T cells – A_450_ medium

Where A_450_ (T cells + Treg) is the absorbance of autologous CD4+CD25- naïve T cells co-cultured with NP73-102-induced CD4+CD25+ T cells, A_450_ Treg is the absorbance of NP73-102-induced CD4+CD25+ T cells cultured alone, A_450_ T cells is the absorbance of autologous CD4+CD25- naïve T cells cultured without induced CD4+CD25+ T cells, and A_450_ medium is the absorbance of culture medium.

**Luciferase assay**

2 ×10^6^ hmDCs were co-transfected with the indicated luciferase reporter plasmid (0.1 μg) under control of the specific promoter containing the gene and the *Renilla* control vector or 1 μg of the control plasmid containing only the luciferase gene together with pNP73-102, pVAX or pANP. 48 h later, the cells were harvested, lysed and luciferase activity was measured using the Dual-Luciferase Reporter Assay System (Promega) and normalized to the *Renilla* control activity according to the product instructions.

**Studies in mice**

Wild type C57BL/6 mice were purchased from the National Cancer Institute (Bethesda, MD, USA). C57BL/6 NPRA-/- knockout mice were provided by Dr. W.R. Gower, Jr. (VA Medical Center, Tampa, FL, USA). Mice were between 6 and 8 weeks of age at the start of the experiments. Bone marrow was removed from femurs and tibias of NPRA-/- or WT mice and adherent cells were cultured in growth medium for 8 days. DCs were purified using CD11c microbeads (Miltenyi Biotec, USA) and incubated with 0.5 mg/ml OVA for 24 hr. OVA-treated DCs (5 x 10^6^ DCs/mouse) were injected i.v. into NPRA-/- mice that had been sensitized by i.p injection of 25 ug of OVA plus 2 mg of alum adjuvant (Imject, Pierce, USA) on day 0 and day 7 and then challenged intranasally on days 14 and 17 with OVA/alum (25 μg/2 mg) On day 18, eight days after adoptive transfer of DCs, mice were euthanatized, bronchoalveolar lavage was done and lungs were removed for sectioning and histopathological staining. Cells were recovered from the BAL fluid, adhered to slides by cytospin, stained with a modified Wright’s stain (Hema3, Fisher, USA) and differential cell counts done under the microscope.
